# Molecular Epidemiological Characterization and Health Burden of Thalassemia in Jiangxi Province, P. R. China

**DOI:** 10.1371/journal.pone.0101505

**Published:** 2014-07-07

**Authors:** Min Lin, Tian-Yu Zhong, Yi-Guo Chen, Jian-Zhong Wang, Jiao-Ren Wu, Fen Lin, Xin Tong, Hui-Tian Yang, Xiao-Mei Hu, Rong Hu, Xiao-Fen Zhan, Hui Yang, Zhao-Yun Luo, Wen-Yu Li, Li-Ye Yang

**Affiliations:** 1 Central Laboratory, Chaozhou Central Hospital Affiliated to Southern Medical University, Chaozhou, Guangdong Province, People's Republic of China; 2 Medical Laboratory, First Affiliated Hospital of Gannan Medical University, Ganzhou, Jiangxi Province, People's Republic of China; 3 Medical Laboratory, Jiangxi Provincial People's Hospital, Nanchang, Jiangxi Province, People's Republic of China; 4 Medical Laboratory, Central Hospital of Iron Industry Limited Corporation of Xinyu, Xinyu, Jiangxi Province, People's Republic of China; University of Central Greece, Greece

## Abstract

**Background:**

Thalassemia is the most common inherited disease in southern China. However, this disorder is usually ignored by Jiangxi provincial health system and government due to lack of epidemiological data.

**Materials and Methods:**

A total of 9489 samples from Hakka Han and Gan-speaking Han in three geographical areas of Jiangxi Province were analyzed for both complete blood cell (CBC) count and reverse dot blot (RDB) gene chip for thalassemia.

**Results:**

1182 cases of suspected thalassemia carriers with microcytosis (MCV<82 fL) were found by CBC count, and were tested by RDB gene chip to reveal a total of 594 mutant chromosomes, including 433 α-thalassemia mutant chromosomes and 172 β-thalassemia mutant chromosomes. Our results indicated a higher prevalence of thalassemia with the heterozygote frequency of 9.49% in southern Jiangxi province, whereas the low frequency was found in middle (3.90%) and northern Jiangxi (2.63%).

**Conclusions:**

Based on the epidemiological data, the estimated numbers of pregnancies in Jiangxi province in which the fetus is at risk for β-thalassemia major or intermedia, Bart's hydrops fetalis and Hb H disease are 34 (95% CI, 16 to 58), 79 (95% CI, 50 to 114) and 39 (95% CI, 27 to 58) per year, respectively. We suggested that prevention network of thalassemia should be established, especially in high prevalent southern Jiangxi (Hakka Han), including establishment of thalassemia database collection, hematological analysis laboratories, genetic counselling clinics, prenatal diagnosis centers and neonatal screening centers.

## Introduction

Thalassemia is inherited as an autosomal recessive disorder characterized by microcytic hypochromic anemia, and a clinical phenotype varying from almost asymptomatic to a lethal hemolytic anemia. It is probably the most common monogenic gene disorder in the world and is especially frequent in tropical and sub-tropical areas such as Mediterranean countries, the Middle East, the Indian subcontinent, southern Far East Africa and Southeast Asia [1], [2]. Approximately 5% of the global populations are carriers of this disorder, with about 60,000 children with α-thalassemia major and 43,917 symptomatic β-thalassemia individuals born annually, the great majority is in the developing world [2], [3]. It creates a major public health problem and social burden to the people in endemic regions [2], [4].

Previous studies have indicated that there was a high population frequency of thalassemia in southern China, mainly in southern regions of the Yangtze River, particularly in the three most southerly provinces of China-Guangdong, Guangxi and Hainan [5]–[8]. With a total area of 1,669,000 square kilometers and a population of 44.62 million (http://www.gztj.gov.cn), Jiangxi province lies on the southern bank of the Yangtze River. It shares a border with Anhui to the north, Zhejiang to the northeast, Fujian to the east, Guangdong to the south, Hunan to the west, and Hubei to the northwest. 99.73% of inhabitants of Jiangxi province are Chinese Han, predominantly Gan-speaking Han and Hakka Han. Two decade have elapsed since a relative low incidence of thalassemia (α-thalassemia: 2.60%; β-thalassemia: 0.18%) was reported by a large scale survey of hemoglobin variants and thalassemia in Jiangxi province (1987), this disorders was usually ignored by health system and government [9]. However, this result was conflicting with the finding of our molecular epidemiological study (2011) for common α-thalassemia and β-thalassemia on border to southern Jiangxi province (Meizhou region of northeast Guangdong) [7]. A high heterozygote frequency of 11.24% on local Hakka population was found in the Meizhou region [7], which suggested that there might be a high-prevalence thalassemia on Hakka population inhabited in southern Jiangxi province.

Here, we perform a large-scale survey of thalassemia in 9489 healthy subjects, which firstly allows a sight into the prevalence and molecular characterization of thalassemia in Jiangxi province. It is a prerequisite for defining a specific policy for carrier screening, genetic counseling and prenatal diagnosis.

## Materials and Methods

### Population Samples

**Figure 1:**
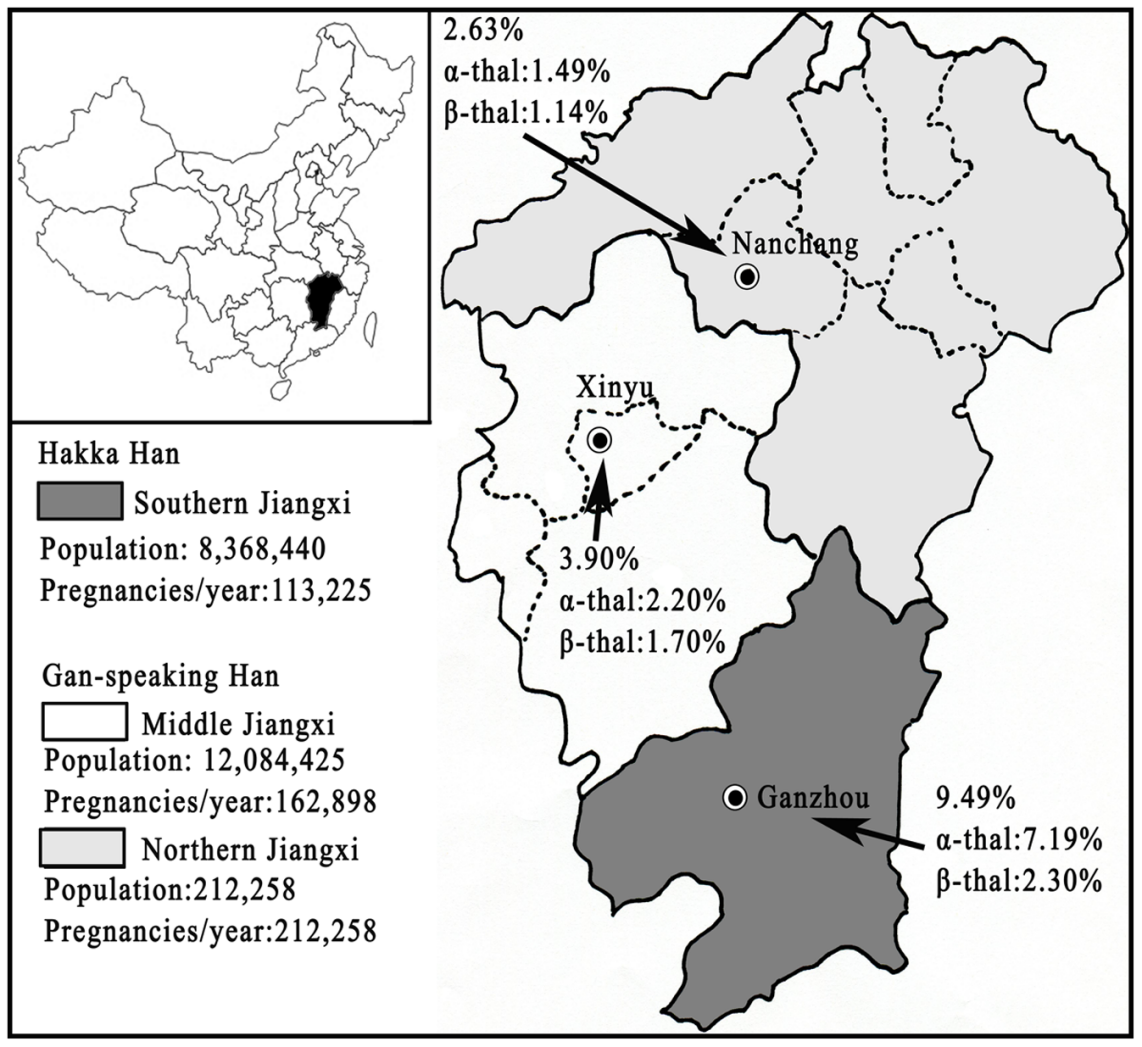
Geographic location of the Jiangxi province and three regional centers of our study.

The study population included 9489 unrelated subjects between August 2011 and November 2011. These subjects visited medical units for routine healthy examination including blood tests, and the discarded blood samples were used for further study. The ages of these subjects ranged from 18 to 70-year-old and about 99% were Jiangxi natives, i.e. Information sheets with nationality, sex, age, dialect, natives or not and written consent forms were available in Chinese to ensure comprehensive understanding of the study objectives, and informed consent was signed or thumb printed by the participants. All studies were approved by the Ethics Committee of First Affiliated Hospital of Gannan Medical University and Ethics Committee of Chaozhou Central Hospital Affiliated to Southern Medical University. Fig. 1 showed the location of Jiangxi province and the three study regions including Ganzhou region (A) (South, 4992 subjects), Xinyu region (B) (Middle, 1002 subjects), Nanchang region (C) (North, 3495 subjects).

### Hematological analysis

These subjects received hematological screening for the presence of thalassemia in Medical Laboratory of the First Affiliated Hospital of Gannan Medical University, Central Hospital of Iron Industry Limited Corporation of Xinyu and Jiangxi Provincial People's Hospital. 2 mL peripheral blood, which was anti-coagulated with 4.0 mg EDTA-K_2_, was obtained from each participant. RBC indices were determined according to standard laboratory procedures by Beckman Coulter-LH750 blood analyzer (Beckman Coulter, Inc., USA). Quality control of complete blood count (CBC) was monitored through participation in the National External Quality Assurance Scheme of China and Randox International Quality Assessment Scheme (RIQAS). Subjects with low red blood cell mean corpuscular volume (MCV) values (<82 fL) were considered possible thalassemia carriers.

### Molecular Diagnosis of α/β-Thalassemia

Genomic DNA of subjects with MCV<82.0 fL were extracted from peripheral blood leukocytes by DNA blood mini kit (QIAGEN China Shanghai Co., Ltd). The DNA concentration was determined by UV spectrophotometer (UNICO Shanghai Instruments Co., Ltd) at the wavelength of 260 nm. The three known α-thalassemia deletions –SEA, −α^3.7^, −α^4.2^, two α-thalassemia mutations [Hb Constant Spring (HBA2: c.427T>C) and Hb Quong Sze (HBA2: c.377T>C)], and 16 known β-thalassemia mutations most commonly seen in the Chinese population (HBB:c.279A>G, HBB: c. −78A>G, HBB:c.250A>C, HBB: c.2T>G, HBB: c.45_46insG, HBB: c.52A>T, HBB: c.79G>A, HBB: c.84_85insC, HBB: c.94delC, HBB: c.92+1G>T HBB: c.92+1G>A, HBB: c.92+5G.C, HBB: c.126_129delCTTT, HBB: c.130G>T, HBB: c.216_217insA, HBB: c.316–197C>T) were analyzed by a commercial thalassemia RDB gene chip (Chaozhou Hybribio Limited Corporation, China). The kit of the gene chip was approved by Chinese SFDA (REG.NO: SFDA (P) 20123400399) [7], [9]. The assay was performed according to the manufacturer's protocol [10].

### Statistical Analysis

Statistical analysis was conducted with SPSS 16.0 statistical software. The prevalence of different thalassemia alleles was calculated from the standard Hardy-Weinberg formula. Data from the three regions in Jiangxi province and previous studies were analyzed by Pearson χ^2^ test. *P*<0.05 was considered statistically different.

## Results

### Population Prevalence and Mutation Spectrum of α- and β-Thalassemia

A total of 9489 blood samples were obtained and analyzed from three regions of Jiangxi province. 1182 cases of microcytosis (MCV<82 fL) were found. The percentage of microcytosis in Ganzhou region, Xinyu region and Nanchang region was 20.15% (1006/4992), 4.69% (47/1002) and 3.69% (129/3495), respectively.

All 1182 microcytosis samples were analyzed by a RDB gene chip for the three known α-thalassemia deletions (–SEA, −α^3.7^, −α^4.2^), two α-thalassemia mutations (Hb Constant Spring and Hb Quong Sze) and 16 known common Chinese β-thalassemia mutations. The results of the survey of population among 9489 blood samples were shown in Table 1. A total of 594 mutant chromosomes were identified, including 433 α-thalassemia mutant chromosomes and 172 β-thalassemia mutant chromosomes. The gene frequency of α-thalassemia and β-thalassemia in various areas of Jiangxi province was shown in Table 2. All these alleles were found with Hardy-Weinberg equilibrium (*P*>0.05).

**Table 1:** Results of the survey of population prevalence of thalassemia among 9489 blood samples in the Jiangxi province.

	Southern Area (Ganzhou, *N* = 4992)		Middle Area (Xinyu, *N* = 1002)		Northern Area (Nanchang, *N* = 3495)	
Variable	Case number	Percentage	Case number	Percentage	Case number	Percentage
α-Thalassemia	348	6.97	22	2.20	48	1.37
α-Thalassemia silent^a^	118	2.36	1	0.10	4	0.11
α-thalassemia trait^b^	229	4.59	21	2.10	42	1.20
Hb H disease	1	0.02			2	0.06
β-thalassemia	115	2.30	17	1.70	40	1.14
β-thalassemia trait	106	2.12	17	1.70	38	1.09
β/α-thalassemia trait	9	0.18			2	0.06
Total	463	9.27	39	3.90	88	2.52

a Including three types of silent heterozygous mutation, −α^3.7^/αα, −α^4.2^/αα, and α^WS^α/αα;

b Including three types of heterozygous mutation (–SEA/αα, α^CS^α/αα, α^QS^α/αα) and a type of homozygous mutation −α^3.7^/−α^3.7^.

**Table 2:** α-, β-thalassaemia alleles and their distribution among the three regions in Jiangxi province.

	Southern Area (Ganzhou, *N* = 4992)		Middle Area (Xinyu, *N* = 1002)		Northern Area (Nanchang, *N* = 3495)	
Variable	Allele number	Percentage	Allele number	Percentage	Allele number	Percentage
α-Thalassemia chromosomes	359^a^	7.19	22^a^	2.20	52^a^	1.49
(–SEA/) Deletion	222	4.45	20	2.00	46	1.32
(−α^3.7^/) Deletion	93	1.86	1	0.10	5	0.14
(−α^4.2^/) Deletion	35	0.70				
HBA2: c.427T>C (Hb CS)	4	0.08				
HBA2: c.377T>C (Hb QS)	5	0.10	1	0.10	1	0.03
β-Thalassemia chromosomes	115^b^	2.30	17	1.70	40^b^	1.14
HBB: c.316–197C>T	45	0.90	6	0.60	19	0.54
HBB: c.126_129delCTTT	35	0.70	5	0.50	13	0.37
HBB: c.−78A>G	21	0.42	3	0.30	3	0.09
HBB: c.52A>T	5	0.10	2	0.20	2	0.06
HBB: c.84_85insC	5	0.10	1	0.10	3	0.09
HBB: c.79G>A	1	0.02				
HBB: c.−79A>G	1	0.02				
HBB: c.130G>T	1	0.02				
HBB: c.216_217insA	1	0.02				
Total	474	9.49	39	3.90	92	2.63

a These numbers include silent heterozygous mutation, α-thalassemia trait, Hb H disease and α-thalassemia compound β-thalassemia;

b These numbers include β-thalassemia trait and α-thalassemia compound β-thalassemia.

Similar to the general geographical distribution of thalassemia in China, the prevalence of thalassemia was higher in the south than in the north. As was shown in Table 2, the heterozygote frequency of thalassemia in Ganzhou region was the highest (9.49%), which bordered Meizhou region of Guangdong province, and approximately 99% of 9 million natives in Ganzhou were Hakka people. Nine samples were compound carriers of α-thalassemia and β-thalassemia, giving a frequency of 0.18% (9/4992) for coincidence of these two common mutations in Ganzhou region (Table 1). The high population prevalence could cause serious health and social problems on Hakka population in Ganzhou region. The prevalence of 3.90% in middle area was a little higher than 2.63% of northern area.

Consistent with previous reports [5]–[7], the Southeast Asian type of deletion (–SEA/) was the most common α-thalassemia mutation, followed by −α^3.7^ deletion and −α^4.2^ deletion. 9 kinds of β-thalassemia genotypes were found in the molecular survey (Table 2). HBB: c.316–197C>T and HBB: c.126_129delCTTT accounted for 64.5% (111/172) of these mutations, again in agreement with previous studies in eastern Guangdong [5], [7].

### The Future Health Burden Prediction

According to the population report of the Jiangxi Provincial government in 2011 [11], annual birth rate and natural growth rate were 13.48% and 0.74%, respectively. The estimated numbers of pregnancies each year in the varied regions in which the fetus would be at risk for β-thalassemia major (TM) or intermedia (TI), Bart's hydrops fetalis, and Hb H disease were shown in Table 3, and 69% (105/152) of thalassemia major or intermedia located in Ganzhou region.

**Table 3:** The future health burden prediction in Jiangxi province.

Variable	Southern area	Middle area	Northern area	Total
Population	8,368,440	12,084,425	15,746,170	44,567,475
Pregnancies/year	113,225	162,898	212,258	488,381
β-thalassemia M-I (95% CI)/year	15 (10–21)	12 (3–25)	7 (3–12)	34 (16–58)
Bart's hydrops fetalis (95% CI)/year	55 (41–68)	16 (5–33)	8 (4–13)	79 (50–114)
Hb H disease (95% CI)/year	35 (26–47)	2 (0–7)	2 (1–4)	39 (27–58)

Data of population and Pregnancies/year was obtained from Web of Jiangxi Provincial government (http://www.gztj.gov.cn); Southern area: Ganzhou region; Middle area: Ji'an region, Pingxiang region, Yichun region and Xinyu region; Northern area: Nanchang region, Jingdezhen region, Wuzhou region, Jiujiang region and Shangrao region. β-thalassemia M-I: β-thalassemia major or intermedia.

## Discussion

We have firstly performed a survey of the prevalence and molecular characteristics of thalassemia in the Jiangxi province of southern China. The natives of Jiangxi province include two Chinese Han subgroups. One-third of the habitants, live in southern part of the province, speak Hakka dialect, are Hakka Han, two-thirds of the habitants live in middle and northern parts of the province, speak Gan dialect, are Gan-speaking Han (Figure 1). Our results indicated a higher prevalence of inherited Hb disorders in southern Jiangxi province: the heterozygote frequency was 9.49%, whereas the low frequency was found in middle (3.90%) and northern Jiangxi (2.63%) (Figure 1). There was a significant difference between southern Hakka Han and Gan-speaking Han. The distribution of thalassemia frequency was similar to the situation of G6PD deficiency in Jiangxi province [12], further confirmed the hypothesis that both human G6PD and the β-globin gene were involved in malaria-protective selection [13], [14].

High prevalence of α/β-thalassemia in the Hakka population of southern Jiangxi province (9.49%) was similar to the situation in Hakka population of Meizhou region in Guangdong province (11.24%, *P* = 0.224), was also similar to average level of Guangdong province (11.07%, *P* = 0.842) [7], [5], but lower than that of Guangxi Zhuang Autonomous Region (24.51%, *P* = 0.000) [6]. Data of this study indicated that thalassemia in Hakka population shared the same set of mutations as the other regions of southern China [5], [7], [8]. α-thalassemia is mainly caused by the Southeast Asian deletion (–SEA), which displays normal hemoglobin or slightly microcytic hypochromic anemia, and therefore results in fetuses with hemoglobin Bart's hydrops fetalis syndrome (absence of all four α-globin genes; commonly as –SEA/–SEA) [15], [16]. HBB: c.316–197C>T, HBB: c.126_129delCTTT, HBB: c.−78A>G and HBB: c.52A>T accounted for 90% β-thalassemia mutations. Not consent with the finding from most regions of southern China (Table 2), HBB: c.316–197C>T was the most common β-thalassemia mutation (39.13%), followed with HBB: c.126_129delCTTT (30.43%), HBB: c.−78A>G (18.26%) and HBB: c.52A>T (4.35%) among the 115 β-thalassemia alleles. The situation that prevalence of HBB: c.316–197C>T mutation was higher than the prevalence of HBB: c.126_129delCTTT mutation, was only found from Hakka inhabited regions and surrounding areas, such as Fujian [17], Taiwan [18], [19], Chaoshan region of eastern Guangdong [20]. According to our knowledge, β-globin gene mutations can be used as genetic markers to study the origin and spread of β-globin gene alleles, revealing historical relationships between populations [21]–[23]. The relative high carrier frequency in the population and the geographical specificity of the mutation suggested that HBB: c.316–197C>T mutation might originate from Hakka population living in Longyan region of Fujian province, Ganzhou region of Jiangxi province and Meizhou region of Guangdong province. This mutation spread to other regions of southern China with immigrated Hakka population in several times because of social unrest, upheaval and invasions [24].

Based on our epidemiological results, thalassemia is a large public health problem in Jiangxi province, especially in Ganzhou region. Ganzhou is a relative poverty-stricken mountainous region in Jiangxi province. The annual per capita disposable income of the urban residents and the annual per capita net income of rural residents are only ¥18,704.7/$3,069.4 and ¥5,301/$869.9 (2012), respectively (Data from the Webpage of the Ganzhou government (http://www.gztj.gov.cn). Children with β-thalassemia major need regular blood transfusions, iron chelation therapy with deferoxamine and even hematopoietic stem cell transplantation. The annual cost of blood transfusions and deferoxamine treatments for one patient with β-thalassemia major is estimated to be about ¥100,000/$15,690 in China [7]. The huge gap of income and medical expenses makes many families extremely poor. Another concern was the danger of Bart's hydrops fetalis for the mother, which resulted from high –SEA thalassemia frequency in Ganzhou region. It could lead to foetal death in utero or soon after birth, and maternal complications such as toxaemia of pregnancy [25]. Therapeutic abortion was suggested for the foetus diagnosed as having this disease. Although ultrasonography provided unambiguous detection of Bart's hydrops fetalis at 18–20 week of pregnancy [26], DNA diagnosis from chorionic villi or amniotic fluid fibroblasts was strongly recommended in Ganzhou region for detecting Hb Bart's hydrops fetalis as early as 10–16 week gestation [27]. Therefore, it needs the local government and the health departments to develop a system of prevention and control program to decrease thalassemia major in the Ganzhou region. As our previous recommendation in Eastern Guangdong [7], [20], we suggested that prevention network of thalassemia should be established in the region, including establishment of a thalassemia database, hematological analysis laboratories, genetic counselling clinics, prenatal diagnosis centers and neonatal screening centers. At the same time, new related policies and laws should be introduced and carried out to prevent thalassemia and help the families with thalassemia major or intermedia.
